# Fire History from Life-History: Determining the Fire Regime that a Plant Community Is Adapted Using Life-Histories

**DOI:** 10.1371/journal.pone.0031544

**Published:** 2012-02-21

**Authors:** Graeme Armstrong, Ben Phillips

**Affiliations:** 1 Research Institute for Environment and Livelihoods, Charles Darwin University, Darwin, Northern Territory, Australia; 2 Centre for Tropical Biodiversity and Climate Change, James Cook University, Townsville, Queensland, Australia; Purdue University, United States of America

## Abstract

Wildfire is a fundamental disturbance process in many ecological communities, and is critical in maintaining the structure of some plant communities. In the past century, changes in global land use practices have led to changes in fire regimes that have radically altered the composition of many plant communities. As the severe biodiversity impacts of inappropriate fire management regimes are recognized, attempts are being made to manage fires within a more ‘natural’ regime. In this aim, the focus has typically been on determining the fire regime to which the community has adapted. Here we take a subtly different approach and focus on the probability of a patch being burnt. We hypothesize that competing sympatric taxa from different plant functional groups are able to coexist due to the stochasticity of the fire regime, which creates opportunities in both time and space that are exploited differentially by each group. We exploit this situation to find the fire probability at which three sympatric grasses, from different functional groups, are able to co-exist. We do this by parameterizing a spatio-temporal simulation model with the life-history strategies of the three species and then search for the fire frequency and scale at which they are able to coexist when in competition. The simulation gives a clear result that these species only coexist across a very narrow range of fire probabilities centred at 0.2. Conversely, fire scale was found only to be important at very large scales. Our work demonstrates the efficacy of using competing sympatric species with different regeneration niches to determine the probability of fire in any given patch. Estimating this probability allows us to construct an expected historical distribution of fire return intervals for the community; a critical resource for managing fire-driven biodiversity in the face of a growing carbon economy and ongoing climate change.

## Introduction

Wildfire is a fundamental disturbance process in many ecological communities across the globe [1], [2]. Indeed, the fire regime is often a critical factor maintaining the structure of plant communities [3], [4] and changes in a fire regime can radically alter community composition [5], [6]. Increasingly, however, we seek to manage fire; either for protection of human lives and assets, to maintain biodiversity, or as part of a growing carbon economy [7], [8]. As the negative biodiversity impacts of inappropriate fire management regimes are increasingly recognized [9], [10] attempts are being made to manage fires within a more ‘natural’ regime. However, it is rarely apparent what, exactly, this means. From an ecological perspective, the critical question in wildfire management is what the fire regime in an area should be: how often, when, and how patchily should an area be burnt so as to maintain or promote current biodiversity values?

Ecologists have sought to answer this question using a variety of techniques that attempt to determine the fire regime to which the community is adapted. One important technique has been the examination of historical records. In Australia, for example, evidence that long-standing, pre-European fire management practices contributed to the demographic structure and geographic range of many vegetation communities [11], [12], [13] has encouraged research into management that emulates the fire regimes imposed by indigenous Australians. While arguably successful in some cases, this approach has not proven broadly applicable, partly due to a lack of resolution in regard to the identity of vegetation communities which came under human influence [14], and partly because of a patchy historical record and loss of indigenous knowledge across most of Australia since European settlement [15], [16]. Analytical techniques have also been employed. For example satellite imagery has become a powerful tool for mapping fire patterns across landscapes [17]. For inferring long-term historical pattern however, it fails simply because satellite imagery does not go far enough back in time. Charcoal deposits can provide longer term data on fire frequency, however they are limited to sites with appropriate depositional conditions and only provide very course frequency resolution inappropriate for ecosystems with very frequent fires, such as savanna which can have a biannual fire return interval [18]. Dendrochronology, the age-dating of trees using tree rings and fire scars also provides quantitative methods for determining fire regimes [19]–[21] but again, the method is not broadly applicable because not all fire prone habitats have tree species with regular cambial growth in which seasonal events are recorded.

A similar technique, applicable to non-woody species, uses knowledge of plant responses to fire (e.g., if the plant is an obligate seeder that only germinates after fire) and data on plant abundance through time to estimate the chronological sequence of fire events over recent history [22]–[23]. This enables the most recent fire intervals to be determined, but again falls far short of making inference about the long-term fire regime to which the community is adapted and is of no use when there is significant germination during the fire interval. [24], [25].

How then, do we go about determining the fire regime to which the community is adapted? The metrics of interest are twofold: fire scale (or patchiness), and fire frequency (or fire return interval). Current understanding is, that on a landscape level, fire can promote diversity by providing spatial and temporal variability in habitat across a landscape, creating a range of niches' through time and space [3]. For example, fast growing and maturing species outcompete slower growing species in areas when the fire return interval occurs before the latter mature and set seed [26]. Similarly, species with greater dispersal abilities are at an advantage when fires are uniformly large [27]. Indeed, species can be classified into different functional groups according to their fire regeneration or persistence niche [28]–[30]. Competing species from different functional groups are able to coexist in a single landscape precisely because they utilise the different spatial and temporal opportunities created when fire removes, or at least inhibits, the growth of competitors. The patchiness of fire, thus, creates high species turnover at the local scale while simultaneously enabling stability at the meta-community scale [3]. The long-term fire regime (frequency and patchiness), then, is that which has allowed the long-term persistence of sympatric taxa with differing regeneration niches.

Thus, if we have data on the life history of several plant species that compete for space in the same landscape, we can ask, using an appropriate model, what is the fire regime that allows these taxa to co-exist over time? If these taxa vary in their regeneration niche, we should rapidly converge on a small list of possible fire regimes that will allow long-term persistence. Here we demonstrate this approach, and its utility for inferring the fire regime to which species have co-adapted, using a simple spatio-temporal simulation model. We parameterized the model with life-history data from three congeneric grasses (*Triodia spp.*), each representing a differing functional group, and then search for the fire regime that allows these three species to co-exist over 200 generations. Rather than modelling fire frequency as a fixed return interval, we treat it as a probabilistic event that affects a patch (and a patch's neighbours if fire scale is large). Treating fire as a probabilistic event allows a distribution of fire return intervals to emerge naturally across the landscape, and it is this distribution which allows competing species to coexist. The models show that the co-existence of these three species can only occur over a very narrow range of fire probabilities but a much broader range of fire scales. This narrow range of probabilities must, therefore, be a key attribute of the long-term historical fire regime of the area.

## Results

**Figure 1:**
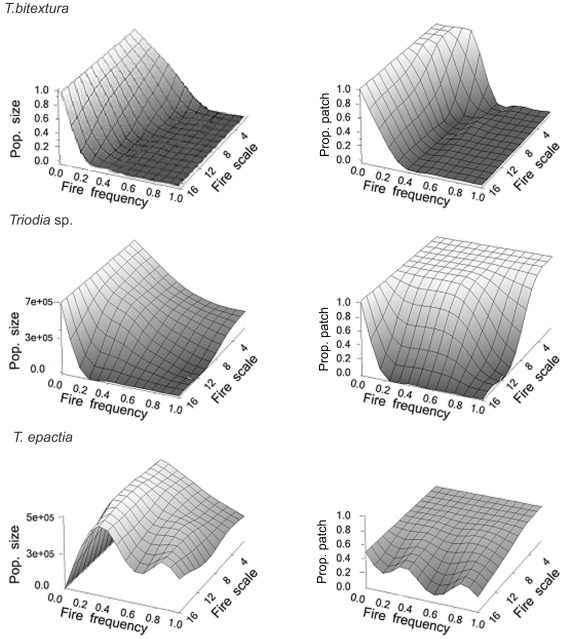
The mean population size (max. 1 million) and proportion of patches occupied (1 = 100%) at different fire frequencies and scales over the entire ‘landscape’ for individual species without competition.

Each of the three species had an individual response to fire probability and scale as a result of differences in their life histories (Fig. 1). The obligate seeder, *T. bitextura*, persisted only at fire probabilities below 0.8 at small scales reducing gradually to approximately 0.4 as scale increased. There is only a small plateau of 100% patch occupancy at the lowest fire frequencies and scales. *Triodia* sp., an obligate seeder, persists at a far greater range of frequencies and scales than *T. bitextura* with total patch occupancy at all frequencies when scale is small. Both population size and occupancy fall away as scale increases and population size is negatively correlated with fire frequency. Conversely, the facultative resprouter, *T. epactia* persists across all frequencies above 0.2 at all scales. Below this frequency persistence is marginal, with very low population size, at medium patch occupancy.

**Figure 2:**
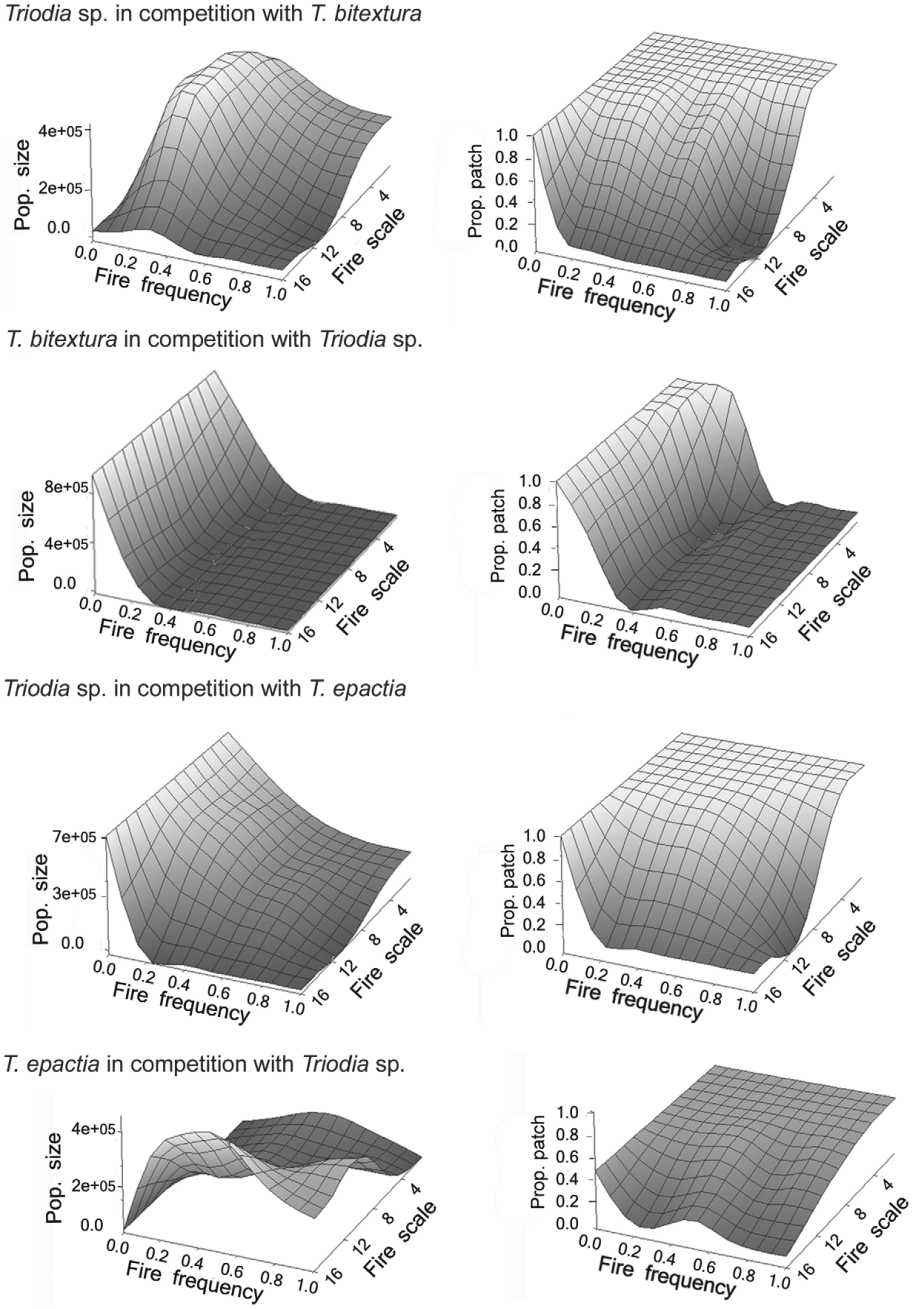
The mean population size and proportion of patches occupied at different fire frequencies and scales over the entire ‘landscape’ when two species are in competition.

Comparison of the individual species responses to fire with those when competition was introduced between pairs (Fig. 2) enabled interspecific interactions to be resolved. *T. bitextura* showed no differences under both competition scenarios indicating the demography of this species is totally determined by fire rather than competition (i.e., it is the competitively superior species). *T. bitextura* inhibited both population size and patch occupancy of *T. epactia* and population size of *Triodia* sp. at low fire probabilities.

**Figure 3:**
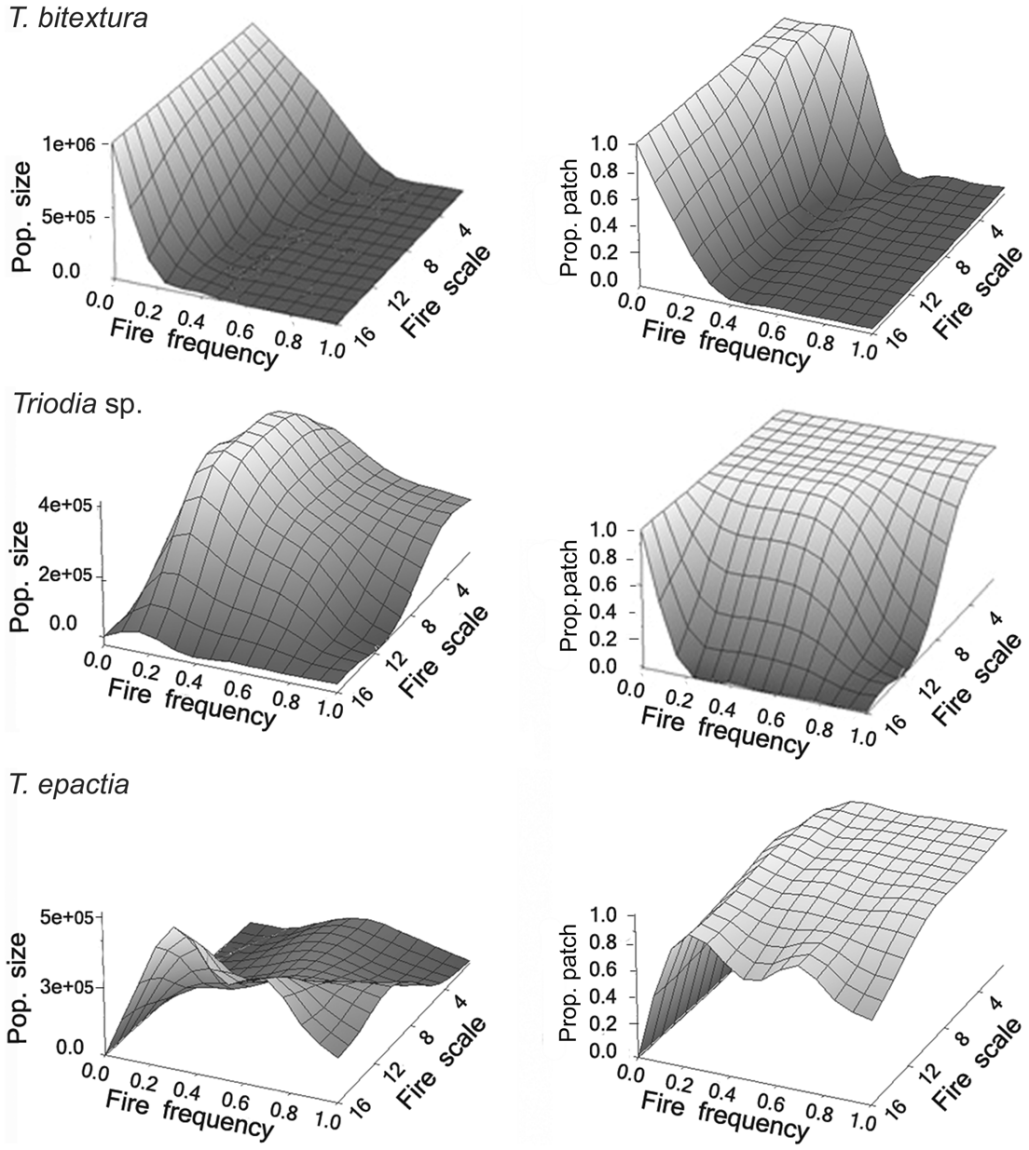
The mean population size and proportion of patches occupied for each species at different fire frequencies and scales over the entire ‘landscape’ with all species in competition.

**Figure 4:**
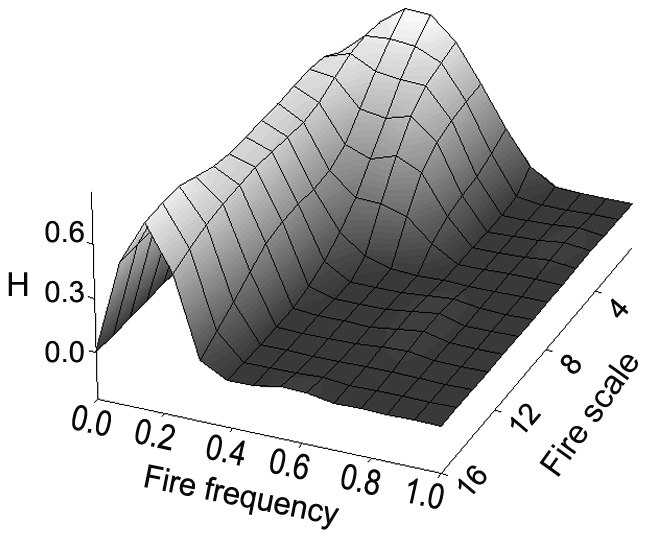
Diversity (Shannon-Wiener index) in those cells in which all three species were present under the full competition model.

**Figure 5:**
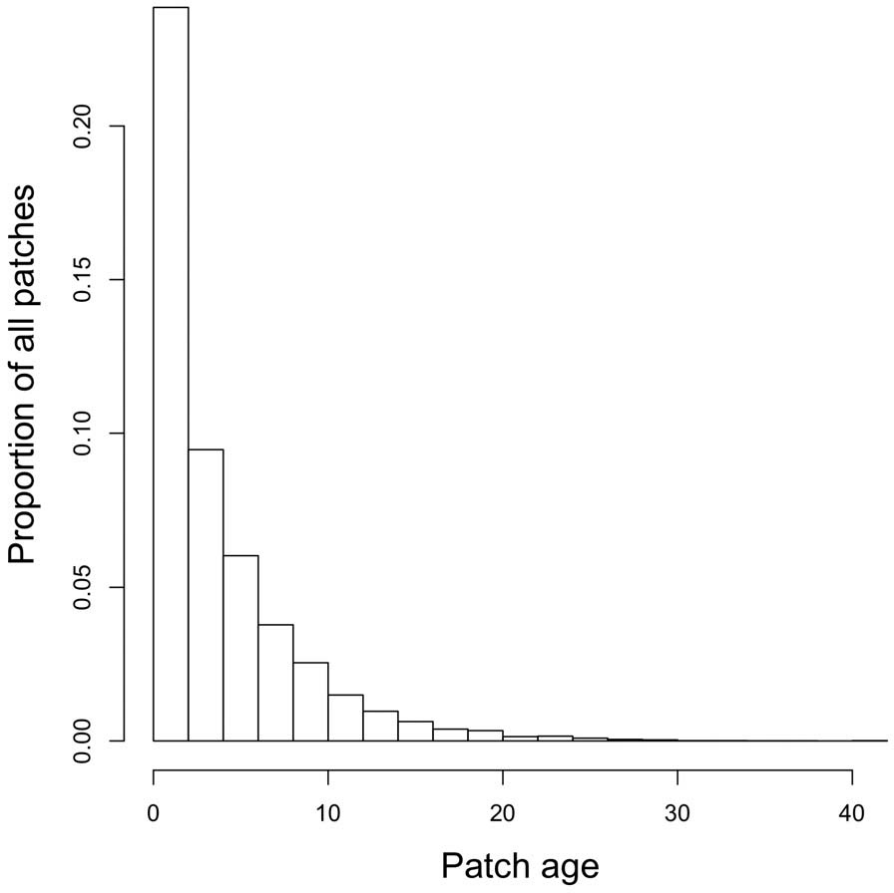
Frequency histogram of age categories (time since last fire measured in years) at a fire frequency of 0.2.

The large range of fire probabilities and scales at which species were able to persist without competition was significantly reduced with competition between pairs and further still when there was competition among all three species (Fig. 3). Visualizing only those cells in which all three species were present (Fig. 4) showed a ridge in mean population diversity centred over fire probability equalling 0.2. The spread, or variability, of the surface about the mean was greater at smaller fire scales, becoming very narrow as the scale increased. When fire is treated as probabilistic, the time since fire over a large number of patches can be described by a negative binomial distribution. When we set the probability of failure in the negative binomial to 0.2 we see a landscape where many patches burnt within the last 5 years (as expected), but with a large number of patches remaining unburnt for considerably longer periods (up to 20 years; Fig. 5).

## Discussion

Our results show that each of the three *Triodia* species has a very different demographic response to fire probability and scale (Fig. 1). This is in line with our classification of these three species into different functional groups, and reflects differences in their regeneration and persistence niches. The obligate seeder, *Triodia* sp., is unable to persist at fire probabilities above 0.2 when fires are large (Fig. 1), indicating that both regeneration time and dispersal ability prevent the landscape persistence of this species under these conditions. *Triodia bitextura*, the obligate resprouter, has a similar response, but appears generally less resilient due to its reduced (i.e., local) dispersal ability. The facultative resprouter, *T. epactia* on the other hand, has a very different response to the other species (Fig. 1), being able to persist at all fire frequencies and scales, although only at very low densities when fire is absent. The low population size of *T. epactia* in the absence of fire is probably due to population senescence: the species lacks clonal reproduction, and fire is needed to remove adults and allow seedling germination.

When three-way competition was introduced into the model (Fig. 3) the demographic response of *T. bitextura* was relatively unchanged compared with the no competition scenario, while there were significant changes for both *Triodia* sp. and *T. epactia*. Thus, *T. bitextura* appears to be the superior competitor, suggesting its demography is totally determined by the fire regime. The other two species, however, are inhibited by competition with *T. bitextura* and/or each other at low fire frequencies. Importantly, when the response surfaces for all species were combined to show only those regions of fire regime parameter space in which all species coexist (Fig. 4) we see a steep-sided ridge centred over a fire probability of 0.2 with greater spread at lower fire scales compared to larger scales. This indicates fire probability has a greater influence on the demographic structure of this community than fire scale. Given that these three species, from different functional groups, are sympatric over an area of approximately 3000 km^2^ in a very old and stable landscape, this narrow range of fire probabilities (around 0.2) is that to which these taxa are most likely to have co-adapted in this landscape.

Long term co-persistence amongst these three species in our model is only possible due to the spatio-temporal structure that fire provides in the landscape with fire probability being the critical factor providing this structure. An average fire probability of 0.2 allows a wide range of fire return intervals to exist across the landscape (Fig. 5). Importantly, this result does not concur with previous fire scar analyses in this region, using remote sensed data [31], in which fire return intervals are typically less than 5 years. Thus, the recent fire frequency across this landscape appears inconsistent with the distribution of fire frequencies we elucidate here and which has enabled the long term persistence of this community. This is a significant observation given the widespread decline of mammals and fire sensitive plant species across northern Australia in recent decades [32]. Inappropriate fire regimes are suspected of playing a major role in these declines, and our results strongly suggest that in our study area, at least, “inappropriate” means “too often”, and perhaps, “too large”.

This study clearly has significant implications for fire management in the study area, where landscape scale fire management is practiced through aerial burning and yearly mapping [33]. Although the consensus among managers in the area is that prior to active management fire occurred too frequently and in too large a patch, our work provides quantitative estimates of how frequent is too frequent, and provides a distribution of fire ages towards which the landscape should move if management is to be effective. Without such quantitative guidance fire management simply remains intuitive. In the same way, our approach is also likely to prove useful for determining the average fire frequency in other plant communities. In making such inference, care will, however, need to be taken to find species from different functional groups that could reasonably be considered to compete for space due to disturbance by fire and not some other niche dimension. This is unlikely to be a problem except in the most changed environments where few original species remain.

Generally, our work demonstrates the efficacy of using competing sympatric species from different regeneration niches' to determine the fire frequency to which they have co-adapted. The approach is, basically, to search for a fire scale and probability that create the correct array of opportunities in space and time for persistence of species from all functional groups. It is this very spatio-temporal habitat structure that enables species from different functional groups to coexist [34], [3], [35], so we can use the fact of coexistence to infer the historical fire regime. We cannot definitively rule out other environmental factors acting as further niche dimensions contributing to the coexistence, or otherwise, of the species in this study, however the evidence that the regeneration niche dominates other potential niche axes is strong in the present case [36]. Further, the species models without interspecific competition demonstrate persistence is limited within definable fire regimes adding weight to the above argument. While we considered fire to be probabilistic in the ‘landscape’, discounting other potential environmental influences on fire behaviour, we feel that any increase in ‘landscape’ heterogeneity would increase the variance of the range of fire probabilities but this would not influence the mean. While this method relies on several independent lines of evidence (life histories of individual species and molecular data regarding population dynamics) it cannot be validated with other data in the way that a charcoal sequence may be correlated to dendrochronological data. However, we feel all methodologies have merit for particular situations and the results presented here are suitable for non-woody ecosystems with intense fire regimes.

The importance of being able to determine the fire regime to which a plant community has adapted cannot be overstated given that the fire regime is a key driver of biodiversity in many systems. This fact, together with the increasing recognition that the global distribution of fire may be altered with climate change [37]–[39], and the increasing demand to use fire as a carbon emission mitigation strategy, means that careful management of fire is in need of quantification. Here we provide a useful way to create fire management benchmarks for biodiversity.

## Methods

### Study area and species

The three species considered in this study, *T. bitextura*, *T. epactia* and *Triodia* sp nov., have widely differing total ranges but grow in sympatry across an 18×140 km region of the southern Kimberley between 17°30′ and 17°40′S. The climate is influenced by the northern monsoon with a mean of 750 mm rainfall occurring in the summer months from October to April and mean annual temperatures ranging from 32C in the summer to 28C in the winter. The landscape is dominated by the highly eroded sandstone of the King Leopold Ranges with basaltic soils and cobbles in the valleys. Fire is the dominant disturbance in this environment with 30% of the landscape burnt annually and a quantitative analysis of satellite data at MWS, covering the last decade, showed a fire return interval of every 2–3 years (Steve Murphy, 2009 unpubl. data). Active fire management to control the season, extent and spatial complexity of fires has recently been introduced [33] following a period of uncontrolled extensive wildfires.

The three target species were chosen for the study because they are dominants in this savanna grassland community and are highly competitive ecological generalists which are distinguished by different post-fire regeneration niches, not by their physiological responses to substrate, moisture or community composition [36], [40]. Each species forms monospecific stands, uncorrelated with edaphic variables, with sharp stand boundaries with little to no intermixing of individuals were different species grow adjacent to each other. Molecular evidence infers they have existed in sympatry in this environment dating back to at least the late Pleistocene [41]. The three study species all rely to differing degrees on fire for regeneration [Armstrong, 2009 unpubl. data]. *Triodia bitextura* is an obligate resprouter that regenerates after fire through a combination of resprouting adults and production of ramets. A small number of stolons are produced between fires which also develop into free living ramets. Virtually no seed is produced and no evidence has been obtained that seedlings contribute to overall regeneration in this species. Adults of *T. epactia*, a facultative resprouter, also resprout after fire, but rely on the germination of seedlings for population growth instead of ramets. *Triodia* sp. an obligate seeder, does not resprout after fire or produce ramets, relying totally on seed germination for population growth. Significant germination only occurs in *T. epactia* and *Triodia* sp. after the removal of adult plants during a fire.

### The model

The spatially explicit model predicts the persistence of each species individually and in competition with the other species across all fire probabilities and a range of fire scales. Stage structured life-history matrices were constructed based on the sexual (seed) and asexual (resprouting and stolons) fecundity of each species and the transition probabilities of each stage class. Life cycle diagrams are shown for each species and the matrices and initial vectors are shown in the Appendix.

The model ‘landscape’, with a carrying capacity of 1 million individuals, approximately represents a 1.6 km^2^ landscape containing 256, 100×100 m cells (‘patches’), each with a carrying capacity of 3906 individuals, based loosely on empirical evidence that a 5×5 m quadrat contains a mean number of 10 individuals. For each model run, across successive proportional fire scales,1, 2, 4, 8 and 16, with 16 being a fire across a 16×16 cell area, which is the size of the entire space, the fire probability ranged from 0–1 at 0.1 increments. Fire scale was therefore initially small but doubled with each successive run until it equaled the entire ‘landscape’.

Each model run was initialized with a vector of the pre-fire, adult condition appropriate for each species. A conservative transition probability of 0.9 was set for all adult stages and ramets to account for observed high survival rates in all species. *Triodia epactia* and *T. bitextura* also have a probability of 0.9 of living indefinitely beyond their climax stages of 3 and 5 years respectively. The number of populations which actually survive beyond 5 years is expected to be small due to the probability of being burnt before this time. Populations of *Triodia* sp. on the other hand are observed to senesce at approximately 4 years old [40] and without fire or dispersal would be expected die out.

We used standard matrix population projection, in which the projection matrix is multiplied by the initial vector, to find the expected population size in each subsequent generation. The resultant population vector was however, modified by three additional (spatial) processes: fire, dispersal and competition. Fire occurred as a stochastic disturbance, random in space. The probability of a fire in any given grid cell in any given generation was determined as the product of two probabilities: the probability of an ignition event, and the conditional probability that an ignition would result in a fire. This latter probability was conditional on whether or not the grid cell had experienced a fire in the previous generation: being set to one where no fire had been experienced, or 0.3 if a fire had been experienced. This is to account for the time needed to establish enough fuel to carry a second fire and is derived by analysing the number of consecutive fires which occurred over 10 years across 100 random points using Landsat imagery (Steve Murphy 2009 unpubl. data). Whether or not a fire actually occurs in a grid cell was then determined by a draw from a binomial distribution for the given frequency.

Dispersal was also modeled as a stochastic process, was scaled by population size and differed between vegetative and seeder species. Vegetative species could only disperse into neighbouring cells (because the amount of seed produced is so negligible that it was considered functionally insignificant), and did so with a probability that scaled with their grid cell population size relative to grid cell carrying capacity. Dispersal distances for *Triodia* seeds are unknown, however seeds and seedlings are observed to fall and grow around the base of adult plants [42] suggesting the majority of seeds travel less than a metre. This would concur with observations for other grasses [43]. However, with maximum annual wind speeds occurring in the summer months (www.bom.gov.au/climate/data/2009) when plants are seeding, and the possibility of animal assisted dispersal, there is a possibility of longer dispersal for at least some seeds. It has been demonstrated that for a range of herbaceous species median seed dispersal distance increased linearly with increasing wind speed but exponentially above the 90^th^ percentile of the seed shadow [44]. This means that with increasing wind speeds the majority of seeds will still fall within metres of the adult plant but 10% will disperse much greater distances. For these reasons dispersal was modelled as global in the seeder species (i.e., a dispersing seed could land anywhere on the landscape) but with a small probability of dispersing long distances. In the model, dispersed seeds germinate in the patch in which they land, without the need for fire-induced germination.

The final stochastic process in our model was competition. Competition assumes that there is limited space in which to grow, and acts to curb population growth in our model. Competition was modeled as a sampling procedure: a survival lottery. When the expected population size of adult plants in a grid cell exceeded the per cell carrying capacity, the final population size of adult plants was determined by a draw from a multinomial distribution with *n* being set to the grid cell carrying capacity and probabilities scaled with the expected number of adults in each age class. Thus, species with a relatively high number of expected recruits into the adult population would, in the absence of disturbance, come to dominate a grid cell.

The model was run in three configurations; 1) each species individually to determine where each species is able to persist across the range of fire variables, 2) with each combination of species pairs to determine which species compete with each other and 3) with all species combined to determine at which fire frequency and scale the community is at maximum diversity (Shannon-Wiener index) reflecting the greatest probability for coexistence. The mean population size and patch occupancy was recorded for the last 5 of the 200 generations of each run and the mean of these taken over 20 replicate runs. The code was written in R (R Development Core Team 2009) [45], shown in the appendix, and the Shannon-Wiener index determined with the vegan package (http://cran.r-project.org/, http://vegan.r-forge.r-project.org/). All the results were plotted against fire scales and frequencies on a 3D spline plot using Tibco S-plus [46]. The frequency of patches of particular ages in the ‘landscape’ i.e. time since last fire (measured in years) was plotted for a fire frequency of 0.2 (Fig. 5).
